# Genetic Architecture and Molecular, Imaging and Prodromic Markers in Dementia with Lewy Bodies: State of the Art, Opportunities and Challenges

**DOI:** 10.3390/ijms22083960

**Published:** 2021-04-12

**Authors:** Romina Combi, Maria Salsone, Chiara Villa, Luigi Ferini-Strambi

**Affiliations:** 1 School of Medicine and Surgery, University of Milano-Bicocca, 20900 Monza, Italy; chiara.villa@unimib.it; 2 Institute of Molecular Bioimaging and Physiology, National Research Council, 20054 Segrate (MI), Italy; salsonemaria@gmail.com; 3 Department of Clinical Neurosciences, Neurology-Sleep Disorder Center, IRCCS San Raffaele Scientific Institute, 20127 Milan, Italy; 4 Department of Clinical Neurosciences, “Vita-Salute” San Raffaele University, 20127 Milan, Italy

**Keywords:** dementia with Lewy bodies, biomarker, genetics, neuroimaging, cerebrospinal fluid, functional imaging, DAT-SCAN, cardiac MIBG scintigraphy, amyloid-PET, tau-PET, FDG-PET

## Abstract

Dementia with Lewy bodies (DLB) is one of the most common causes of dementia and belongs to the group of α-synucleinopathies. Due to its clinical overlap with other neurodegenerative disorders and its high clinical heterogeneity, the clinical differential diagnosis of DLB from other similar disorders is often difficult and it is frequently underdiagnosed. Moreover, its genetic etiology has been studied only recently due to the unavailability of large cohorts with a certain diagnosis and shows genetic heterogeneity with a rare contribution of pathogenic mutations and relatively common risk factors. The rapid increase in the reported cases of DLB highlights the need for an easy, efficient and accurate diagnosis of the disease in its initial stages in order to halt or delay the progression. The currently used diagnostic methods proposed by the International DLB consortium rely on a list of criteria that comprises both clinical observations and the use of biomarkers. Herein, we summarize the up-to-now reported knowledge on the genetic architecture of DLB and discuss the use of prodromal biomarkers as well as recent promising candidates from alternative body fluids and new imaging techniques.

## 1. Introduction

Dementia with Lewy bodies (DLB) was introduced as an independent entity only 20 years ago due to its clinical overlap with other neurodegenerative disorders and to its clinical heterogeneity. At present, it is considered one of the most common causes of dementia, affecting from 0% to 5% of individuals in the general population and accounting for 0% to 30.5% of dementia cases [1]. Through a systematic review of previously reported studies, DLB incidence was estimated to range from 0.5 to 1.6 per 1000 person-years in the general population [1]. From a clinical point of view, DLB mostly occurs in the elderly, with an average age at presentation of 75 years old [2] and shows several symptoms, including cognitive fluctuations with altered attention and alertness, spontaneous parkinsonism, recurrent visual hallucinations, visuo-spatial dysfunctions and rapid eye movement (REM) sleep behavior disorder (RBD) [3]. Sometimes, patients also reported neuroleptic sensitivity and transient loss of consciousness [4]. The clinical differential diagnosis of DLB from other similar disorders is often difficult and the recognition of the disease was originally possible only by post-mortem neuropathological studies on brain autopsy tissues of patients with a clinical history of senile dementia, demonstrating a typical Lewy Bodies (LB) distribution. The histopathological analysis revealed in fact a progressive α-synuclein (α-syn) accumulation and aggregation in Lewy bodies in the brainstem, limbic and neocortical regions [5,6]. The α-syn pathology in the cortical area is similar to the one reported for Parkinson’s Disease (PD) but, interestingly, it was observed in DLB patients also at the beginning of the disease, while in PD the α-syn pathology starts in the dorsal motor nucleus of the vagus [7,8].

Autopsy studies demonstrated that DLB is frequently underdiagnosed and that a portion (ranging from 33 to 50%) of carefully clinically diagnosed AD patients shows LB pathology [9,10]. Moreover, a recent study showed variability in DLB clinical diagnosis rates (2.4−5.9%) between clinicians in the UK, demonstrating a difficulty in properly diagnosing the disease and highlighting the need of an improvement both in diagnostic methods and in the use of specific biomarkers [11]. One of the difficulties in the assessment of a correct diagnosis of DLB is represented by the existing relationship between this disease and Parkinson’s Disease Dementia (PDD). The two disorders show an overlap of symptoms but they are also different in some aspects. As an example, while DLB is typically associated with a cognitive impairment with mild or absent extrapyramidal motor features, PDD shows prominent extrapyramidal motor features.

With the aim of overcoming difficulties in differential diagnoses, the International DLB consortium proposed a list of diagnostic criteria [5] that takes advantage of several clinical observations as well as of biomarker studies. The consortium grouped these features in the following categories: core clinical features, supportive clinical features, indicative biomarkers and supportive biomarkers [5]. The latter distinction of biomarkers lies in their diagnostic specificity as well as in the supportive evidence reported for them.

In this review, we firstly summarize the genetic and epigenetic architecture of DLB, supporting the idea that it is a complex multifactorial disorder with genetic heterogeneity where causative mutations explain a small percentage of cases while in the majority of patients the disease onset is due to an interaction of common variants and environmental factors. Then we will present the up-to-now reported biomarkers useful in DLB differential diagnosis.

Presenting altogether the genetic architecture of DLB as well as molecular and imaging biomarkers will allow us to have, for the first time, a comprehensive view on DLB pathogenesis, shading light on its biological correlates in terms of protein misfolding and aggregation (also considering their different levels and distribution in biological fluids or tissues) and on the best strategies to discriminate this disease from the other overlapping proteinopathies causing neurodegeneration. This could help clinicians in choosing the best strategy for the diagnostic process of each patient.

## 2. Genetics and Epigenetic Architecture of DLB

The knowledge of the genetic etiology of DLB is still limited despite the disease frequency due to the unavailability of large cohorts with a certain diagnosis. Commonly, DLB is a sporadic, late-onset disease. However, a number of families were reported showing a genetic inheritance of the disease, and siblings of DLB probands were reported to have a significantly higher risk of DLB than siblings of AD patients [12]. These families were studied deeply to search for DLB candidate loci and these analyses supported the involvement of genes already involved in PD and AD. These genes include *SNCA*, *SNCB*, *LRRK2*, *GBA*, *APP*, *PSEN1*, *PSEN2*, *PGRN*, and *PRNP* [13]. Nonetheless, families with highly penetrant alleles are very rare.

A first genome-wide association study (GWAS) on DLB [14] provided evidence of an important role (i.e., 36% of heritability) of genetic factors in the pathogenesis of the disease independently from its sporadic or familial origin. More recently, the same group updated their heritability estimate (which is now evaluated 59.9%) by reanalyzing data from the GWAS in DLB and indicated that DLB genetic risk factors are probably independent from known AD and PD risk variants [15]. Genetics is not the only risk factor influencing DLB disease onset, as demonstrated by the low rate of concordance in monozygotic twins [16]. A role of additional unknown factors (these being of environmental or epigenetic origins, or both) in the etiopathogenesis of DLB must exist. Few studies are available specifically analyzing these factors in DLB, while several reports are available for other dementias.

The GWAS study, besides identifying variants in several genes already implicated in neurodegenerative disorders, confirmed the disease association with variants in three of the above-listed candidate genes (*APOE*, *SNCA*, and *GBA*) [14]. Associations with these 3 genes have all been reproduced by independent groups [17,18,19], thus confirming their importance in the disease diagnosis. All the other reported genes are not so strongly supported by the literature data and, in some cases, their detection in DLB could be due to a misdiagnosis and overlapping among different kinds of dementias. However, very recently, a whole exome sequencing of about 1118 Caucasian DLB patients, which was focused on genes already known to cause monogenic neurodegenerative diseases, documented a rarity of previously reported pathogenic mutations, suggesting an insubstantial genetic overlap among diseases [20]. The study found rare variants of unknown significance and a pathogenic mutation in the *GRN* that was detected in a single patient [20].

In the following paragraphs, we summarize reported genetic risk factors identified in DLB, including both rare variants and common variants. Some of them appear to have a causal role in the pathogenesis of the disease while others seem to be modulators of the disease risk. Overall DLB results in a genetically heterogeneous disease with a rare contribution of pathogenic mutation and relatively common risk factors. 

Moreover, we summarized the identified epigenetic factors involved in DLB.

### 2.1. Genetic Factors

#### 2.1.1. Synuclein Genes

One of the well-established and first-identified genes associated with DLB is *SNCA* encoding the α-syn, a small protein playing an important role in synaptic transmission [21]. Two rare point mutations (p.Glu46Lys and p.Ala53Thr) have been reported in DLB cases [14,22,23]. The p.Glu46Lys was firstly detected in a familial case of DLB with autosomal dominant inheritance [22], while the p.Ala53Thr was first identified in an Italian pedigree with individuals showing phenotypic variability during the disease course and reporting PD and a range of dementia severity [24]. Moreover, in a large pedigree, an *SNCA* gene triplication was reported and was associated with a wide spectrum of phenotypes ranging from PD to DLB [25]. These very rare causative mutations show expression variability and result, even within the same family, in a phenotypic spectrum ranging from DLB to different other forms of dementia (PD, PDD, Multisystem Atrophy (MSA), frontotemporal Dementia (FTD)) [26]. 

Different non-causative variants have been so far identified as genetic risk factors in the *SNCA* gene in DLB patients. Among them, rs974711 and rs134893877 were identified by analyzing 43 tagging single nucleotide polymorphisms (SNPs) of the *SNCA* gene [27]. The study confirmed the previously reported existence of distinct association profiles toward the 3′ or the 5′ of the gene for Parkinson’s and/or dementia, respectively [18,27]. Another SNP significantly associated with DLB in the *SNCA* is rs7681440 [14]. Apart from the latter SNP, the reported associations have not been replicated yet [28].

Alpha-synuclein is a member of a large family of proteins including β- and γ-synucleins. Few studies are available evaluating the association between genes encoding these related synucleins and DLB. In particular, two rare missense mutations (p.Pro123His and p.Val70Met) were detected in the *SNCB* gene of DLB cases [29], while SNPs in *SNCG* where associated with sporadic Diffuse Lewy Body Disease (DLBD) [30]. The pathogenicity of the reported variants has been, however, questioned due to a lack of replication or, in the case of the mutations in *SNCB,* due to the lack of segregation with the disease [29].

#### 2.1.2. *APOE*

Another well-established genetic factor contributing to the risk of DLB is the ε4 allele of *APOE*, which is known for its association with AD. This is one of the most studied variants in DLB, with 25 studies reported, the majority of which show a positive association between the ε4 allele and a higher risk of DLB [28]. However, some authors have questioned the possibility that the identified association could not be due to independent stimulation of α-synuclein pathology, but is more likely caused by the effects on amyloid or tau pathologies, and studies on animal models have been performed to elucidate the involved mechanism, suggesting a possible direct role of *APOE* ε4 in promoting synucleinopathy [31,32,33]. 

The *APOE* ε2 allele was reported to be associated with a decreased risk of DLB [32,34,35,36] and a single study also reported a similar association between DLB and the ε3 allele [37].

Interestingly, different papers reported an association of short survival span in DLB and a specific *APOE* genotype [38,39], thus suggesting the importance of genetic testing to assess the prognosis of the disease. 

#### 2.1.3. *GBA*

*GBA* encoding the lysosomal glucosylceramidase beta (GCase) is a well-known gene causing Gaucher disease and is associated with PD [40]. However, heterozygous *GBA* mutations (with a reported mutation frequency ranging from 4 to 31% due to different cohort selection and origins [41,42]) as well as variants in the same gene were reported as predisposing factors for DLB [14,19,43,44,45,46]. DLB cases were reported to be 8 times more likely to be carriers of mutation in this gene than healthy controls [19], a higher risk in respect to the one found for PD patients [47].

As far as the prognostic value of *GBA* mutations goes, the study performed on Ashkenazi Jewish showed an earlier disease onset and more severe cognitive and motor impairment in individuals with these mutations [42].

The knowledge on the presence of *GBA* mutations may be helpful in differential diagnosis of DLB vs. PD. Two studies reported a higher mutation rate in DLB patients with respect to PD patients and, in particular, they found a higher presence of these mutations in patients with pure neocortical LB pathology than in those with mixed AD/LB pathology or with brainstem LB [41,43].

#### 2.1.4. *PSEN1/2*

Presenilins are components of the γ-secretase complex that plays an important role in the amyloid processing. Several rare mutations were reported both in the *PSEN1* and in *PSEN2* genes in AD as well as in DLB [39,46,48,49,50]. However, other researchers reported negative findings by studying the association of DLB and presenilin1 [51]. Due to these contrasting results and to the overlapping with AD, these rare variants could not be useful in DLB differential diagnosis. 

#### 2.1.5. *MAPT*

Different studies reported a significant association of the *MAPT* gene, encoding microtubule-associated tau protein, and DLB [52,53,54]. In particular, a positive association was reported for the so-called *MAPT* H1 haplotype that was also associated with PDD [55]. However, a more recent study was not able to replicate these results [14], thus excluding the use of *MAPT* variants as possible diagnostic or prognostic markers.

#### 2.1.6. *APP*

Rare mutations in the *APP* gene encoding the amyloid precursor protein are known to be linked to AD and, more recently, they have been associated with DLB [20,56,57,58]. Due to the observed heterogeneity and typical overlap of phenotypes within the same family where these mutations segregated, these mutations result in not being useful as biomarkers for DLB, not allowing discrimination between DLB and AD.

#### 2.1.7. Additional Variants in DLB

Several variants with no replication of unknown pathogenetic significance have so far been detected in few papers by exome studies [20,46], GWAS and other researches. All well-established, as well as not replicated variants and haplotypes linked to DLB are reported in Table 1.

Among them, Fujioka and colleagues [74] identified a mutation in the *EIF4G* gene (encoding the Eukaryotic translation initiation factor 4G), previously associated with PD, in two unrelated pedigrees with suggested DLB.

Rare variants in the *LPR10* gene were reported in DLB pedigrees, also showing individuals affected by PD [67].

The *SCARB2* gene, encoding the scavenger receptor class B member 2, was identified as a risk modifier for DLB by the above-mentioned first GWAS study [14,18], emphasizing the role of the lysosomal pathway. However, it has not yet been replicated by other studies.

Data reported in the same study were suggestive of an association between rs7314908 in *CNTN1* and DLB; however, a new study with improved statistical power is necessary [14]. 

A variant in *PRNP* was also reported [71], but its role in the disease etiology was questioned due to its detection in healthy controls [75]. Moreover, no replication was reported.

Recently, a non-synonymous variant in the *PLCG2* gene has been reported as a factor that confers protection from DLB as well as from other dementias [76]. 

A recent study analyzed the role of copy number variations (CNVs) and reported several CNVs significantly associated with DLB, some of which were absent in either databases or healthy controls [61]. Among them, 5 CNVs were detected using a case/control approach (Table 1). Additional CNVs were identified in genes already associated with neurodegenerative disorders or in well-established DLB genes (only those with a p-value available are included in Table 1) [61]. 

### 2.2. Epigenetics Factors

Since its introduction, epigenetics has changed its definition and its understanding is further changing. In general, epigenetics includes changes that affect gene activity and expression, but that are not based within the DNA sequence. Among these epigenetic changes, the most studied are DNA methylation and histone acetylation, two mechanisms important in regulating gene expression through a change in chromatin compactness.

Epigenetic modifications represent promising biomarkers for the diagnosis of neurodegenerative disease [77]. Few studies have analyzed DNA methylation related to DLB, while no studies on histone acetylation are available. In particular, a study compared the methylation state of the *SNCA* intron 1 in DLB vs. healthy controls and showed a reduction in methylation in DLB patients [78]. Other studies evaluated the methylation state of CpG islands located either downstream to exon 1 or in the exon 4 of the *APOE*, demonstrating a hypomethylation in DLB brain in the former case but not in the latter [79,80]. On the contrary, a hypermethylation of three CpG islands in the *DRD2* gene encoding the dopamine receptor D2 was reported in DLB patients [81]. A comprehensive review summarized studies on DNA methylome in different dementias and the overall analyses suggested the existence of common methylation patterns in a number of molecular pathways [82]. Interestingly, common patterns of promoter methylation were detected in DLB and PD in genes involved in different pathways [82,83,84] and some of them could differentiate PD and DLB patients from healthy controls. For a complete description of the up-to-now reported epigenetic studies on Lewy Body Disorders (LBD) and a discussion on their possible use as biomarkers and therapeutic targets highlighting advantages and limits, see Desplats et al. [84].

## 3. DLB Biomarkers in Biological Fluids

### 3.1. CSF Biomarkers

Although cerebrospinal fluid (CSF) sampling by lumbar puncture is an invasive outpatient procedure, CSF still represents the promising source of biomarkers for central nervous system (CNS) disorders. Given its direct contact with the brain parenchyma, CSF potentially reflects the cerebral pathophysiological processes occurring during the disease. Unlike AD, there is no specific diagnostic biomarkers for DLB. Some candidate molecules have been investigated as promising DLB biomarkers, but the results have been often questionable. Among them, the core AD biomarkers in CSF, i.e., the 42 amino acid isoform of amyloid beta (Aβ42), phosphorylated tau (p-tau) and total tau (t-tau) [85], have been largely explored also for DLB as Aβ plaques and neurofibrillary tangles (NTFs) were detected in approximately 40% of patients with PD, PDD and DLB at autopsy [86]. The relevant role of AD pathology in DLB has been confirmed by a multicenter study including a large cohort of patients affected by PD, PDD and DLB. A CSF AD profile with low Aβ42 levels combined with high levels of p-tau and/or t-tau was found in almost 25% of DLB patients, compared with only 3% and 9% of patients with PD and PDD, respectively. Interestingly, DLB patients displaying a CSF AD profile performed worse on Mini-Mental State Examination (MMSE) compared to those with normal CSF [87]. Given the clinical and pathological overlap with other neurodegenerative disorders, core AD biomarkers in CSF have also been investigated as potential differential diagnosis tools in DLB patients. In a cohort of cognitively normal individuals and patients clinically diagnosed as AD, PD, PDD and DLB, Parnetti et al. [87] showed the lowest mean concentration of Aβ42 in CSF from DLB patients that negatively correlated with dementia duration. Moreover, they also displayed lower mean CSF t-tau levels than AD group but higher than PD, PDD patients or normal control subjects with a positive association with dementia severity assessed by MMSE. Concerning p-tau, its levels were significantly increased only in AD patients, in line with a higher presence of NTFs. Collectively, these data suggested that Aβ42 combined with tau in CSF may be useful in differentiating DLB from PD and PPD, but not from AD [88,89]. In contrast with these results, some authors found significantly reduced Aβ42 and elevated t-tau levels in AD with respect to DLB patients [90]. Conversely, other studies did not report any differences in CSF Aβ42 levels of DLB compared to AD with tau proteins higher in the AD group [91,92]. A possible explanation for these contrasting results may be related to the disease stage. In this regard, a retrospective study performed on a large cohort of patients with AD and DLB at prodromal and demented stages showed that the majority of DLB patients display a normal CSF profile at the prodromal stage but reduced CSF Aβ42 levels at a later stage reaching those of AD patients. Independently of disease stage, CSF p-tau and t-tau were confirmed to be the reliable biomarkers to discriminate AD from DLB [93]. The accuracy of differential diagnosis between AD and other dementia syndromes, including DLB, was further improved by the Aβ42/Aβ40 ratio in CSF [94]. Unlike AD, DLB patients at the prodromal stage showed a CSF Aβ42/Aβ40 ratio similar to controls [93]. Other authors demonstrated that the Aβ42/Aβ38 ratio was the strongest biomarker in discriminating AD from DLB, suggesting a possible role of Aβ38 in the differential diagnosis [95]. 

As α-syn represents the major constituent of LB recognized as the main histopathological hallmarks of DLB, several studies addressed the discriminating value of different α-syn species in CSF (i.e., total-α-syn (t-α-syn), oligomeric-α-syn (o-α-syn), phosphorylated-α-syn at serine 129 (pSer129-α-syn)), although the results have been controversial. In a large cohort of patients affected by various forms of dementia, Spies and colleagues did not find any significant differences in CSF α-syn levels among the groups [96]. On the other hand, some authors reported lower α-syn levels in DLB compared to AD and other dementia cases [92,97], while other ones found the opposite results with a significant increase of CSF α-syn levels in DLB patients with respect to the AD group and controls [98], or versus PD, PDD and controls [99]. Although α-syn alone seems not to be a reliable biomarker of DLB, its clinical value may be improved in combination with AD biomarkers. Llorens et al. found that the t-tau/t-α-syn ratio is able to accurately discriminate controls from DLB patients compared to single t-α-syn and t-tau [100]. Another study confirmed the hypothesis that α-syn species combined with AD biomarkers may represent good predictors for DLB. In particular, a combination consisting of Aβ42, tau proteins, t-α-syn, o-α-syn, age and sex, was useful to differentiate DLB from normal control subjects with a good sensitivity and specificity while no differences were found in CSF pSer129-α-syn among the groups [101]. Interestingly, the recent application of two ultrasensitive protein amplification assays, namely Protein-Misfolding Cyclic Amplification (PMCA) and the Real-Time Quaking-Induced Conversion (RT-QuIC) has been successful for the detection of α-syn aggregates in biological fluids of patients with synucleinopathies, mainly in the CSF. Although these techniques have been performed only in a limited number of studies, the results seem to be promising in the identification, with a high sensitivity and specificity of individuals developing synucleinopathies at the preclinical stage [102].

In addition to the classical AD biomarkers and α-syn species, some candidate molecules have been proposed as potential DLB biomarkers, mostly related with disease pathogenesis or neurodegeneration processes. Among them, 3-methoxy-4-hydroxyphenylethyleneglycol (MHPG), the major metabolite of the neurotransmitter norepinephrine in the brain which aids the identification of central noradrenergic activity, has been investigated as a potential CSF marker for DLB owing to the fact that dysfunctions in dopaminergic and serotonergic pathways are crucial in the development of dementia in addition to motor symptoms [103,104]. Using liquid chromatography, two independent studies reported reduced MHPG levels in the CSF of DLB patients compared to AD [105,106], which is congruent with post-mortem observations of decreased MHPG concentrations in different brain regions of DLB patients in respect to AD [104]. This is also in line with earlier evidence of low levels of norepinephrine in the putamen and neocortex in DLB [107]. Interestingly, the addition of MHPG to the core AD biomarkers in CSF was related to the clinical diagnosis of DLB and to a high accuracy in differentiating DLB from AD. The MHPG discriminative power between these two disorders in CSF was further confirmed using a panel combining the levels of core AD biomarkers with monoamine and their relative metabolites, although the authors found high levels of MHPG in DLB/PDD patients compared to other dementia groups [108]. A possible explanation for these contrasting results may be probably related to differences in the study population characteristics and/or psychotropic medication in the DLB/PDD group [108]. 

It has been shown that lysosomal dysfunction, typically associated with intracellular protein accumulation, represents an early event in the pathogenetic processes, leading to synucleinopathies [109]. Moreover, in PD and DLB patients, mutations in the *GBA* gene represent the common genetic risk factor for both disorders [19]. Given these considerations, some authors investigated the CSF GCase activity in a small Italian cohort of patients suffering from AD and FTD, as well as in age-matched healthy controls [110]. A significant decrease in CSF GCase activity was found only in DLB patients compared to the other dementia groups and controls, thus suggesting a specific impairment of this enzyme in synucleinopathies [110]. This evidence was further confirmed by the reduced GCase activity and its mRNA levels in the substantia nigra of DLB and PD brains [111].

Another potential CSF biomarker for DLB is represented by fatty acid binding protein 3 (FABP3), a cytosolic protein linked to neurodegeneration with a role in neuronal activity and synapse formation [112]. In a large cohort of patients affected by AD, PD, PDD and DLB, Chiasserini and collaborators found significantly increased CSF FABP3 levels in both AD and DLB patients compared to PD and healthy controls. Intriguingly, the use of p-tau in combination with FABP3 better improved the diagnostic accuracy in discriminating DLB from AD [97].

A recent study based on a high-throughput proteomic approach aimed to identify novel candidate CSF biomarkers for DLB [113]. In a well-characterized discovery cohort, 69 proteins were found to be differentially expressed in DLB with respect to controls. Data were then replicated in an independent cohort of patients with related neurodegenerative disorders, confirming six promising DLB biomarkers, namely VGF, SCG2, NPTX2, NPTXR, PDYN and PCSK1N. Using machine learning, the authors also identified a panel comprising VGF, SCG2 and PDYN that was capable of better differentiating DLB from other related neurodegenerative disorders with a good specificity and sensitivity. Moreover, low levels of all the identified candidates, except for PCSK1N, were also associated with a more severe cognitive decline [113].

### 3.2. Blood Biomarkers

Drawing blood is a much less invasive procedure than lumbar puncture to collect CSF from patients and is suitable for repeated sampling or screening at early disease stages, therefore some potential molecules have also been explored in this biological fluid, such as peripheral biomarkers for DLB. As expected, the majority of studies focused on α-syn detection in blood but with conflicting results. Laske et al. found decreased α-syn serum levels in DLB compared to AD patients and controls. The authors hypothesized that low α-syn in serum may be the result of the accumulation of α-syn in the brain [114]. In contrast, another study detected higher levels of o-α-syn in plasma from both LBD and PD patients than controls subjects [115]. Moreover, Bougea and colleagues reported that patients with PD, PDD and DLB have significantly more elevated values of serum α-syn in comparison with healthy individuals, excluding its reliability in the differential diagnosis among synucleinopathies [99]. A significant increase of α-syn levels were also found in whole blood from DLB patients with good specificity and sensitivity with respect to AD, vascular dementia (VaD) and healthy subjects. However, high α-syn levels in the DLB group were not found within the erythrocyte fraction, suggesting an increase of this protein in plasma or other blood cellular components, like platelets [116]. In the attempt to identify peripheral biomarkers for disease diagnosis or progression, some authors recently analyzed the differential expression of five *SNCA* transcripts in blood of DLB and PD patients. The results revealed a decrease in all *SNCA* transcripts that positively correlated with disease duration in DLB. Intriguingly, one of the transcripts, namely *SNCAtv3*, showed low levels in early DLB and high levels in early PD, making it a promising biomarker to discriminate these two disorders at the prodromal stages [117]. 

In addition to CSF, MHPG has also been suggested as a potential peripheral biomarker for DLB. Interestingly, this molecule is able to cross both the blood–brain (BBB) and CSF–blood barriers [118]; therefore, alterations in its plasma levels may represent an indicator of noradrenergic dysfunction. Some authors have found low concentrations of MHPG in plasma from DLB patients compared to all the other groups, including AD, FTD and controls. In combination with the CSF AD core biomarkers, they argued that not only CSF MHPG, but also serum MHPG strongly improved the differential diagnosis between DLB and AD, although this panel was not successful in discriminating synucleinopathies [108]. Interestingly, another study revealed that levels of serum MHPG, noradrenaline (NA), MHPG/NA ratio together with MHPG in CSF are able to differentiate Lewy body disorder subtypes from healthy individuals, further suggesting an impairment in the noradrenergic system in these diseases [119].

Among other candidate biomarkers for DLB, heart-type fatty acid binding protein (H-FABP) was shown to be promising for differentiation between dementias. As mentioned above, FABPs represent a family of small intracellular proteins involved in the transport of fatty acid in cytosol and is released into the extracellular space after cellular damage [120]. Increased levels of serum H-FABP were detected in DLB patients compared to other dementia types and non-demented controls [121,122]. Interestingly, the combination of CSF t-tau, serum and CSF H-FABP, as well as the ratio of serum H-FABP to CSF t-tau, could help in the differential diagnosis between AD and DLB [122]. 

## 4. miRNA Expression Profiling

A recent approach to detect new DLB biomarkers in biological samples relies on the study of the expression profile of miRNAs. These are small non-protein-encoded-single-chain RNA molecules contributing to the post-transcriptional protein expression regulation in the cell and playing a crucial role in several processes [123]. Altered expression profiles of miRNAs have been associated with different forms of dementias and neurodegenerative diseases by several studies [124,125]. miRNAs could be easily isolated from different biological samples (e.g., plasma, serum, CSF, saliva, urine) and, being tissue-specific, they might be used as prognostic and diagnostic biomarkers also for psychiatric and neurodegenerative diseases [126,127,128]. 

A comprehensive analysis, by means of several machine learning approaches, of miRNA expression data of 169 Japanese DLB serum samples was reported in 2019 [129]. The study identified 180 miRNAs contributing to the risk prediction model. Among them, 7 showed the highest feature importance and they all target genes belonging to the DHA signaling pathway already associated with the disease [130]. 

The study of miRNAs in DLB has become a new strategy aimed at identifying biomarkers able to discriminate the disease from other dementias (mainly AD and PD). Several studies have been so far reported studying miRNAs purified by different biological samples. Gamez-Valero et al. [131], by profiling the whole miRNA transcriptome from platelet belonging to DLB, AD, and PD patients as well as to healthy controls (HC), identified a 7-miRNA biosignature with a high potential for the differential diagnosis between DLB and AD. In particular, they detected 3 groups of miRNAs, each specifically deregulated in one of the analyzed disorders. The DLB-specific group was made by: Hsa-miR-142-3p and hsa-miR-150-5p, showing reduced expression compared to HC; the same two miRNAs, together with hsa-miR-25-3p, hsa-let-7d-5p, hsa-miR-146a-5p, hsa-miR-132-5p, and hsa-miR-26b-5p, were decreased in respect to AD, while hsa-miR-26b-5p and hsa-miR-150-5p were down-regulated compared to PD. The authors reported no differences in miRNA levels in whole blood, thus suggesting that the identified signature of platelet-specific miRNA deregulation could be related to the pathogenesis of DLB.

In a pilot study, considering a cohort of 8 DLB patients and 10 HC, differences in gene expression profiles in anterior cingulate cortex were reported revealing a list of 14 candidate miRNAs for DLB [132].

Analogously, Nelson et al. [133] performed expression profiling of miRNAs purified by autoptic frozen tissue from aged human brains of DLB patients. In particular, the authors focused their work on the anterior cingulate and primary motor cortical tissues. The study identified several miRNAs differently expressed in the two regions and suggested that this could be the cause of the different vulnerability of brain regions to LBD pathology.

Very recently, several authors focused their attention in the search of miRNA DLB biomarkers by studying the miRNA transcriptome in extracellular vesicles (EVs), which are known to play a role in different processes of the CNS [134,135,136]. Several EVs have been already associated with neurodegenerative diseases [137,138], but only a few papers were reported treating DLB. In one of them, Gamez-Valeo and colleagues [139], by comparing each other the expression profiles of 238 miRNAs purified from plasma-EVs, identified no significant differences between DLB cases and age-matched HCs. Otherwise, they detected 6 miRNAs (hsa-miR-21-5p, hsa-miR-451a, hsa-miR-23a-3p, hsa-let-7i-5p, hsa-miR-126-3p, and hsa-miR-151a-3p) down-regulated in AD patients compared to DLB or HC individuals. In particular, the authors suggested a possible use of hsa-miR-21-5p and hsa-miR-451a, already reported as AD biomarkers [140,141,142], in the DLB vs. AD differential diagnosis. For a comprehensive review of circulating exosomes’ miRNAs differently expressed in dementias and useful in differential diagnosis see [143]. 

## 5. Skin Biomarkers of DLB

In 2017, Donadio and colleagues [144] investigated the pSer129-α-syn and its deposits in skin biopsies to explore the role of peripheral nerves in DLB and to evaluate the possibility of using them in differentiating DLB from other dementias. In DLB patients, they observed abnormal deposits with a typical proximal-distal gradient with a maximum level at the cervical site. Moreover, they reported a higher frequency of pSer129-α-syn deposits in the skin nerves of patients, showing more autonomic symptoms than in those not experiencing these symptoms. Very interestingly, the authors observed different patterns of pSer129-α-syn deposits between DLB and nonsynucleinopathy dementia (NSD) patients: deposits were found in autonomic skin nerve fibers of all DLB while in no cases of NSD. This suggests a possible use of this marker to perform differential diagnosis of different forms of dementia. The use of skin biopsies to test pSer129-α-syn deposits would have several advantages in the clinical practice: it is not expensive, is not as uncomfortable for the patient and the biopsy site is not important due to the observed widespread positivity in DLB with autonomic symptoms. The potential use of this marker, however, must be confirmed by additional studies.

Limits and methodologies used to perform studies on α-syn deposits in skin biopsies for synucleinopathies diagnosis were analyzed in a recent review [145]. An alternative and new approach in the skin biopsies analysis could be the use of RT-QuIC, already reported as successful in studying skin pSer129-α-syn seeding activity in PD [146,147].

## 6. Prodromic Biomarkers of DLB

Prodromal DLB is a term used to identify the pre-dementia clinical stage, a condition in which there may be signs/symptoms indicating that DLB will develop over the time. These mainly include cognitive deficits but also variable clinical features such as motor symptoms and signs, sleep disorders as RBD, autonomic dysfunctions, and neuropsychiatric disturbances [148,149,150]. This is possible, since in the neurodegenerative disorders there is a long period of several decades between these first clinical manifestations and the onset of frank dementia. Considering the clinical relevance of this crucial period, researchers and clinicians felt the need to characterize the prodromal DLB stage with formal diagnostic criteria. In this context, McKeith et al. have recently identified three-onset presentations for the prodromal phase of DLB: (i) mild cognitive (MCI); (ii) delirium-onset; (iii) psychiatric-onset presentations. Their purpose was to determine whether there is currently sufficient evidence to justify the development of diagnostic criteria for each of these categories [148]. Despite sufficient findings to propose formal criteria only for MCI-Lewy bodies disease, authors suggest that it is important to continue to investigate also the delirium-onset and psychiatric-onset presentations [148]. This is even more true as the identification of the prodromal stage allows early intervention while the pathologic process is circumscribed before clinical symptoms become debilitating, and to anticipate treatments known to be effective in this chronic disease [148]. Having this in mind, here we will summarize and discuss: first, how prodromal DLB stages are usually present and second, which clinical biomarkers can be used to identify this precious condition early. The clinical relevance of this evidence for public health consists not only in limiting the progression of DLB but also in improving the quality of life for patients and their care givers.

In order to answer the first question, RBD is one of the core clinical symptoms characteristic of frank-developed DLB. It is a REM sleep parasomnia characterized by abnormal dream enactment behaviour during REM sleep, in which the normal paralysis is lost and thus the patients “act out” their dreams [148,151,152,153]. It is accompanied and confirmed by the loss of muscle atonia during REM sleep (REM sleep without atonia or RSWA) on polysomnography (PSG) registration [148]. The idiopathic (or alternatively “isolated”) form of RBD (iRBD) [153], defined by the absence of ongoing neurologic conditions, is highly associated with the synucleinopathies such as PD and DLB. There is robust evidence for iRBD as a valid biomarker to early identify Lewy bodies-related conditions and to strongly predict the latter transition in defined neurodegenerative disorders. A post-mortem study demonstrated that 80% from the iRBD cohort developed a defined LB neurodegenerative syndrome over the time and, among these, about 30% were DLB [154]. In line with this observation, a recent meta-analysis performed on 51 longitudinal studies revealed that the risk of developing neurodegenerative diseases was 33.5% at 5 years’ follow-up, 82.4% at 10.5 years and 96.6% at 14 years. Among LBD, about 25% of iRBD patients converted to DLB [155]. Misfolded α-synuclein in the CSF of patients with iRBD has been detected by RT-QuIC with high diagnostic accuracy (sensitivity and specificity equal to 90%). Considering that α-synuclein positivity is associated with increased risk of subsequent diagnosis of PD and DLB, its detection might represent a potential prodromal marker of these neurodegenerative disorders and a precious target in the neuroprotective trials [156]. Taken together, these findings suggest that iRBD may be a candidate for the study of early events and progression of this prodromal phase, and to test disease-modifying strategies to slow or stop the neurodegenerative process [154].

With respect to the second question, it is urgent to understand how to identify these clinical biomarkers of the prodromal DLB stage. Indeed, the identification of predictors of dementia and Parkinsonism in iRBD patients has been a matter of interesting investigation. Postuma et al., in a large multicentre cohort of iRBD patients, assessed the neurodegenerative disease risk and predictors of neurodegeneration combining prospective follow-up data from 24 centres of the International RBD Study Group [151]. Among the predictor markers tested including sex, daytime somnolence, insomnia, restless legs syndrome, sleep apnoea, urinary dysfunction, orthostatic symptoms, depression, anxiety, or hyper-echogenicity on substantia nigra ultrasound, only cognitive variables were different at baseline between those converting to primary dementia versus Parkinsonism [151]. This evidence was in line with other multicentre studies confirming that the clinical feature able to predict dementia in iRBD patients is the neuropsychological performance [157]. In details, these authors found that Trail Making Test (part B) best detected early prodromal dementia stages, whereas the best tests for monitoring changes over time were verbal fluency and verbal episodic learning tests. According to their results, prodromal DLB is detectable up to 6 years before onset, since cognitive performance changes and memory deficits were strongly associated with later development of dementia [157]. Finally, in a recent review, some authors summarized evidences regarding the neuropsychological, electrophysiological and neuroimaging biomarkers in iRBD patients when the neurodegeneration is yet to come [158]. Longitudinal studies showed that impaired executive functions, rather than visuospatial abilities, augmented the conversion risk. Cortical slowdown during wake and REM sleep suggested the presence of an ongoing neurodegenerative process paralleled by cognitive decline. Among neuroimaging findings, widely discussed in the following section, the impairment of both nigrostriatal dopaminergic and noradrenergic systems might be a good marker to identify patients at a higher risk of short-term conversion [158]. In conclusion, iRBD is a valid clinical biomarker of the prodromal DLB stage. It is a fascinating phenomenon in the middle of a pathogenetic process in which many neuropathological alterations have already begun and others have yet to occur. In this precious pre-clinical window, the development of novel biomarkers strictly related to the pathological process able to capture the phenoconversion into neurodegenerative disorders represents the most important challenge.

## 7. Imaging Biomarkers of DLB

The role of neuroimaging in evaluating the mechanisms underlying the pathogenesis of DLB has been widely documented. In the last few years, sophisticated and highly sensitive techniques have been developed in order to assess the pathological processes occurring in different regions involved in DLB-neurodegeneration. Researchers have investigated not only the brain structural/functional alterations, but have also explored the relationships with clinical manifestations. An emerging research topic is molecular imaging, a powerful tool to detect in vivo brain function and support an accurate ante-mortem diagnosis. Indeed, novel molecular imaging radiotracers have been applied to identify abnormal proteins aggregation, altered neurotransmitter system pathways and brain metabolism in DLB-neurodegeneration. Key molecular imaging techniques include positron emission tomography (PET) with different radiotracers, single-photon emission computed tomography (SPECT) with ligands such as [123I]-N-fluoropropyl-2β-carbomethoxy-3β-(4-iodophenyl), nortropane (FP-CIT, DAT-SCAN) and [123I]-metaiodobenzylguanidine (MIBG) cardiac scintigraphy. Despite neuroimaging tools having an advanced diagnosis, stratification and monitoring of the disease, new questions emerge in the scenery of imaging modalities including which techniques are able to capture the neuropathological processes. 

Since DLB-neurodegeneration might vary across the different accumulation of abnormal proteins, functional/structural damage in the brain and mechanisms leading to neuroinflammation, here we considered the neuroimaging biomarkers within three sections to target respectively: (i) the neural damage; (ii) the brain tissue damage; (iii) the neuroinflammation. This approach might be of great interest, considering that the same abnormal proteins, although different in the burden and pattern of distribution, could result in different proteinopathies such as DLB, AD, FTD and PDD. Additionally, the new frontier of multimodal imaging should be used to better characterize this chronic neurodegenerative disorder. Emphasis is placed on the early identification of neuroimaging biomarkers for the opportunity that this creates for neuroprotective treatments and specific disease-modifying therapies. Table 2 summarizes the main neuroimaging biomarkers and imaging tools used in DLB studies.

### 7.1. Neuroimaging Biomarkers to Assess the Neural Damage 

The goal of molecular imaging has been to directly or indirectly bind the neuropathological aggregates in the brain and extra-cranial tissues according to the neuropathological model of the disease progression. In this context, the ideal radiotracer should have a selective and high affinity for the aggregates, for example synuclein rather than amyloid and tau, high penetration in the brain and fast clearance. In this section, we will summarize and discuss the main molecular imaging used to identify the pathological neuroaggregates in the DLB-neurodegeneration.

#### 7.1.1. α-Synuclein Aggregates

The turning point of DLB imaging will come when the researchers and clinicians will have the possibility to image in vivo the α-synuclein aggregation. The development of an α-synuclein radiotracer will have the same impact on DLB as the β-amyloid and tau radiotracers have had for AD neuropathology [159]. Braak et al. proposed a staging system to characterize LBDs neuropathologically, suggesting that the temporal sequence of synuclein containing inclusion bodies represents the key feature to differentiate within the spectrum of LBDs [160]. According to this temporal progression model, Lewy bodies accumulation from the medulla (Stage 1) to more rostral structures such as the sublaterodorsal nucleus (involved in developing RBD, Stage 2) and to the substantia nigra (involved in developing PD, Stages 3–4) and the neocortex (DLB, Stages 5–6) [160,161]. Detecting α-syn could mean taking an instant picture of the disease. On the other hand, the development of tracers represents, to date, the greatest challenge in the field of movement disorders. Among the possible reasons for this are: the low concentrations of protein compared to β-amyloid, the predominant intracellular location of α-syn, the existence of multiple protein isoforms, and off-target binding [162,163]. Despite these difficulties, a number of compounds, in particular small polyaromatic molecules, have now been identified with modest characteristics for binding to synuclein fibrils. The compounds [18F]BF227 and [18F]WC-58 are among these: the former has a high affinity for amyloid and a low affinity for α-sin in brain tissues [164], while the latter has promising selectivity and affinity for synthetic fibrils but a slow clearance [165]. Considering that the direct biomarkers of α-sin aggregate load could be problematic, molecular imaging with neuroimaging indirect biomarkers are currently used to determine its effects on the brain by studying noradrenergic and dopaminergic dysfunctions. Thus, reduced cardiac MIBG scintigraphy and DAT-SPECT uptakes have been included as supportive neuroimaging biomarkers in the revised DLB consensus criteria [3]. According to these criteria, indicative biomarkers allow the generation of two diagnostic categories of probable and possible DLB. When one or more of these is found in association with core clinical features, a probable DLB should be diagnosed. In contrast, in the absence of core clinical features, it is possible to classify the dementia as possible DLB [3].

- Noradrenergic System

The MIBG ligand is an analogue of the sympathomimetic amine guanethidine, widely used to determine the location, integrity, and function of postganglionic noradrenergic neurons [166]. It allows in vivo the measurement of cardiac uptake relative to a mediastinal reference uptake calculated as a heart/mediastinum ratio (H/M ratio) [167]. A reduced cardiac MIBG uptake reflects the loss of postganglionic presynaptic cardiac sympathetic nerve endings, due to the presence of Lewy body-related disorders. Lewy body lesions, including Lewy neurites, are encountered in extracranial tissues, notably in autonomic ganglia [168,169,170]. To support this, some authors have recently found a close relationship between cardiac denervation and α-syn deposition in the autonomic nerves [171]. Additionally, this pathological condition can be present even before the sympathetic ganglia involvement, in an early phase of the DLB neuropathological process [172,173], suggesting a role as preclinical biomarker of Lewy bodies-related conditions [174].

Severe cardiac noradrenergic post-ganglionic denervation is a common feature in DLB but not in AD, thus offering a potential system for a biological diagnostic marker [175]. Single center studies have reported excellent ranges of sensitivity (94−100%) and specificity (87−100%) values in distinguishing DLB from AD [167,176,177,178]. Moreover, in a cross-sectional multicenter study, some authors have re-evaluated the diagnostic accuracy of MIBG scintigraphy performed at baseline for differentiating probable DLB from probable AD after 3-year follow-up [179]. These findings confirmed high correlation between abnormal cardiac sympathetic activity at baseline and a final clinical diagnosis of probable DLB [179]. Other authors, in a recent meta-analysis, used a regression model to analyze the delayed mean H/M ratio of MIBG uptake for distinguishing 2 diagnostic clusters: (1) PD, DLB, and RBD; (2) healthy controls and AD, MSA, progressive supranuclear palsy, VaD, and FTD patients. The scintigraphic tool accurately distinguished among these diagnostic clusters with 94% sensitivity and 91% specificity [180].

Cardiac MIBG scintigraphy, however, is not only useful to support the differential diagnosis among dementias, but it is also a biomarker of clinical DLB conversion. Haruiko et al. recently compared the usefulness of brain perfusion SPECT and cardiac MIBG scintigraphy in predicting the conversion of possible DLB to probable DLB. According to their results, the areas under the ROC curves for 123I-MIBG based on the earl H/M ratio, delayed H/M ratio, and washout rate were 0.935, 0.936, and 0.884, respectively, while for occipital/cerebellum and occipital/striatum cortex ratios detected on a SPECT scan were 0.591 and 0.585, respectively. The authors concluded suggesting that cardiac MIBG scintigraphy may be a good predictor of the future conversion from possible to probable DLB [181]. Despite great enthusiasm for this technique, however, potential confounding factors including comorbidities (ischemic heart disease, heart failure, diabetes mellitus, peripheral neuropathies) and medicines (tricyclic antidepressants and sympathomimetics) could produce artefacts that should be considered by researchers/clinicians for correctly interpreting MIBG images and results.

- Dopaminergic System

123 I-FP-CIT is a ligand able to selectively bind the presynaptic dopamine transporter, widely used to analyze the integrity of the nigrostriatal projection pathway. In particular, 123 I-FP-CIT-DAT-SPECT has been applied in a large number of trials in order to identify in vivo the loss of dopamine transporters in the striatum of patients with presynaptic parkinsonism [182,183,184,185,186]. The dopaminergic neuron degeneration is visible on DAT-SPECT imaging as a reduction of radiopharmaceutical-specific uptake. Thus, the rationale of this approach is to capture the nigrostriatal degeneration occurring in DLB-neurodegeneration.

Similarly, to cardiac MIBG scintigraphy, the utility of DAT-SPECT imaging in distinguishing DLB from AD is well established, with a sensitivity equal to 78% and a specificity of 90%, respectively [3,187]. It is also worth highlighting that when parkinsonism is the only core clinical feature of DLB in a patient with dementia, reduced DAT uptake as indicative biomarkers warrants a probable DLB diagnosis [3]. Nonetheless, the specificity of DAT imaging can decrease when the differential diagnosis is between DLB and neurodegenerative disorders, such as vascular parkinsonism or FTD, also characterized by loss of the nigrostriatal integrity [188,189]. Considering the limitations of DAT-SPECT imaging as a stand-alone tool, the combined use with cardiac MIBG scintigraphy should be encouraged [187,190,191]. Few cross-sectional studies have compared the diagnostic value of the two scintigraphic tools [178,192]. It has been reported that both techniques have similar sensitivity for detecting DLB, but the latter appears to be more specific for excluding non-DLB dementias [193]. With this in mind, some authors have determined whether the combined use was superior to using either modality alone for diagnosing suspected DLB [194]. They also calculated a combined index and evaluated its diagnostic ability. In the diagnosis of DLB, DAT-SPECT, MIBG myocardial scintigraphy and combined index may be reliable indices. In particular, MIBG myocardial scintigraphy was the specific modality for an accurate diagnosis of DLB. Understanding the effectiveness and limits of both scintigraphies and using both properly could lead to a more accurate diagnosis and better treatment [194].

DAT SPECT imaging proved useful in characterizing the clinical phenotype in DLB patients. Shimizu et al. have recently assessed the correlation between clinical symptoms and regional low DAT uptake in the striatum of patients with DLB [182]. Their main results demonstrated that when comparing the DLB patients with or without fluctuations, visual hallucinations, or RBD, there were no significant differences in DAT uptake in any regions of the striatum. In contrast, DLB patients with parkinsonism had significantly lower DAT uptake in the entire striatum, entire putamen, and anterior putamen compared to DLB patients without parkinsonism. Finally, they found a weak but significant correlation between severity of parkinsonism and DAT uptake in entire regions of the striatum in patients with DLB. The authors concluded that only parkinsonism was associated with a reduction in striatal DAT uptake in their patients with DLB.

#### 7.1.2. β-Amyloid and Tau Aggregates

Despite the main histopathological hallmark of DLB-neurodegeneration being the α-syn contained in the LBs, β-amyloid and tau aggregates may coexist with α-syn in clinically diagnosed DLB patients, suggesting a spectrum of a mixed neuropathology [195,196]. Cumulating in vivo evidence of β-amyloid and tau aggregates have been frequently observed in amyloid-PET and tau-PET studies of probable DLB patients, supported by post-mortem data. Additionally, there is a general consensus that β-amyloid and tau, whether present, may interact synergistically with other neuropathologic processes to aggravate the picture of DLB. Clarifying the role of these aggregates to the DLB pathophysiology represents a key target for developing novel therapeutic treatments. The presence of β-amyloid and tau co-pathologies in the brain of DLB patients, however, raises some clinical questions. First, from a neuropathological point of view, it is of great interest to identify whether β-amyloid and tau proteins accumulate alone or in combination and which imaging tools should be applied to capture this intriguing phenomenon. Second, from a clinical point of view, it is needed to investigate the implications in the clinical phenotype of these proteinopathies. This is crucial considering that a subset of DLB patients also exhibits a mixed neuropathology; is the presence of additional neuroaggregates to α-sin associated with atypical rather than typical forms of DLB? Finally, from a therapeutic point of view, it should be investigated how AD- and tau-related pathology influences the timing and the neurodegenerative process; what is the impact of co-pathologies on the severity and progression of the disease?

Numerous post-mortem evidence has shown that AD-related pathology is frequent in DLB [197,198,199,200,201]. Among neuropathologic features of AD pathology, however, diffuse plaques (DPs), comprised primarily of Aβ42, are typically abundant in these patients, also occurring in the absence of NFT–tau pathology, while neuritic plaques (NPs) comprised primarily of Aβ40 have mainly observed in AD patients [202]. In vivo-PET imaging is in line with these evidences, reporting elevated Aβ in more than half of the DLB patients [203]. In this context, 11CPittsburgh compound B (PiB) is one of the most widely investigated Aβ PET ligands. The development and validation of the PiB for in vivo imaging of α-amyloid deposition has had the most impact on the in vivo diagnosis of AD and its preclinical stages [159]. The clinical relevance of PiB is due to the fact that it exclusively binds to the β-pleated sheet of the amyloid protein in the cortical gray matter and the Aβ deposits in the vessel walls [159]. Kantarci et al. [202] investigated the pathologic correlates of PiB uptake on PET in cases with antemortem diagnosis of probable DLB at autopsy. They found that Aβ pathology relatively spared the occipital lobes in cases with mixed pathology and LBD compared to cases with AD without LBD and that lower PiB uptake accurately distinguishes cases with LBD from cases with AD or mixed pathology. The severity of diffuse Aβ pathology is the primary contributor to elevated PiB uptake in DLB [202]. Some authors found that the cortical amyloid burden was higher in the DLB group than in the PDD group, comparable to the AD group [204]. In detail, in the DLB group, amyloid deposition in the parietal (lateral and precuneus)/posterior cingulate region correlated to visuospatial impairment and striatal PiB retention with less impaired motor function. These findings suggest that whether it presents, β-amyloid may contribute selectively to the cognitive impairment of DLB and to the timing of dementia relative to the motor signs of parkinsonism [204]. AD-related pathology appears also to influence the rate of brain atrophy over time measured with MRI. A faster atrophy, in particular in the medial temporal lobe, has been reported by several reports in DLB patients when compared to those without concomitant pathology [205,206,207,208,209]. Overall, this evidence demonstrates that when amyloid is present, it mainly contributes to temporal lobar atrophy, with a pattern of brain deposition and atrophy resembling that observed in AD [210,211,212].

Few investigations have assessed the longitudinal pathological AD-related changes in DLB patients. Nedelska et al. [213] determined the trajectory of β-amyloid accumulation in a cohort of 35 consecutive patients with DLB from the Mayo Clinic Alzheimer Disease Research Center. They also investigated the associations of β-amyloid accumulation with measures of clinical and cognitive decline over time in DLB. Higher baseline PiB and change in PiB were associated with a more rapid clinical and cognitive decline over time. Measuring the change in PiB may have implications for designing anti–β-amyloid randomized clinical trials for DLB patients [213]. Sarro et al. [207] investigated the associations between baseline PIB binding and the longitudinal rates of grey matter atrophy in a cohort of clinically diagnosed patients with dementia with Lewy bodies, who were consecutively recruited to the Mayo Clinic Alzheimer’s Disease Research Centre. They identified significant associations between greater baseline PiB standard unit value ratios and greater grey matter loss over time in the posterior cingulate gyrus, lateral and medial temporal lobe, and occipital lobe as well as caudate and putamen nuclei, after adjusting for age. Higher β-amyloid deposition at baseline is predictive of faster neurodegeneration in the cortex and also in the striatum and predicts a faster clinical decline over time in patients with DLB [207].

The contribution of tau pathology in the normal aging and in the course of DLB-neurodegeneration has been difficult to evaluate. This is due to two main factors: brain tau-concentrations are much lower than those of β-amyloid and configurations of the tau abnormal protein are multiple. To further complicate the picture, the distribution of tau pathology in DLB seems to follow an atypical pattern when compared to patients with typical AD [214]. The influence of comorbid tau on DLB clinical phenotypes, cognitive functions and brain imaging is well-documented. A key contribution to the tau-imaging has been obtained with radioligand [18F] AV-1451 based on its high affinity, selectivity and favorable kinetics [215,216]. Recent postmortem evidences have confirmed that this tracer is able to bind strongly to tau in neurofibrillary tangles and neurites without binding Aβ and, above all, it does not bind α-synuclein aggregates or Lewy bodies [214,217]. In DLB-neurodegeneration, the tau burden is intermediate between healthy controls and AD, although the level of concomitant amyloid may be highly influential [218,219,220]. Indeed, DLB patients exhibit a high burden of tau in specific regions, including occipital and posterior temporoparietal regions when compared to healthy controls, but less than that observed in AD patients. In line with this, two previous converging studies have reported greater [18F]-AV1451 uptake in the inferior temporal gyrus, precuneus, and occipital regions in DLB compared to the controls [221,222]. Recently, Mak et al., have partially confirmed these results [223] by observing that [18F]-AV1451 tracer uptake was not elevated in DLB relative to normal aging [223]. Minimal tau binding, however, was associated with cognitive impairment, highlighting the key role of tau to underlie cognitive dysfunction in DLB [223]. The medial temporal lobe [18F]-AV1451 BPND also distinguished DLB from AD patients, confirming the high discriminative performance of tau-imaging in the differential diagnosis between these two chronic neurodegenerative disorders.

Multimodal imaging approaches able to capture both proteinopathies (β-amyloid and tau) should be encouraged. In a multi-center cohort of 417 patients with DLB, some authors tested the hypothesis that amyloid-β and tau biomarkers positivity increased with age, was modified by APOE genotype and sex, and that there were synergistic associations with the clinical phenotype [219]. Positivity on β–amyloid (A+) and tau (T+) biomarkers was determined by CSF amyloid-β 1-42 and phosphorylated tau in the European cohort, and Pittsburgh compound-B and AV-1451 positron emission tomography in the Mayo Clinic cohort. They found that AD–tau related pathologic changes are common in DLB and were selectively associated with the clinical phenotype. Indeed, while amyloid-β was associated with cognitive impairment, tau pathology was associated with a lower frequency of clinical features of DLB [216]. Additionally, two multimodal PET-imaging studies should be cited [224,225]. In the first study, [18F] AV-1451 and [11C] PiB were used to assess in vivo tau and β-amyloid protein aggregates, respectively, in clinically probable DLB compared to the age-matched AD patients [221]. Patients with probable DLB had greater AV-1451 uptake in the posterior temporoparietal and occipital cortex compared to clinically normal controls, and in probable DLB the uptake in these regions correlated with global cortical PiB uptake. Medial temporal lobe AV-1451 uptake also distinguished probable DLB from AD [221]. In the second study, DLB PET-scan was compared to the control subjects. According to their results, cortical [18F] AV-1451 uptake was highly variable and greater than in controls, particularly in the inferior temporal gyrus (ITG) and precuneus. Of note, elevated cortical [18F] AV-1451 binding was observed in 4/17 LBD cases with low cortical [11C]PiB retention. A greater [18F] AV-1451 uptake in the ITG and precuneus was associated with increased cognitive impairment, as measured with the MMSE score [222]. Finally, postmortem evidence has shown that the coexistent mixed pathology can result in a less typical presentation of DLB core clinical features with a lower frequency of recurrent visual hallucinations, parkinsonism, RBD and fluctuating cognition and a more severe disease course [224,225,226,227,228,229].

**Figure 1:**
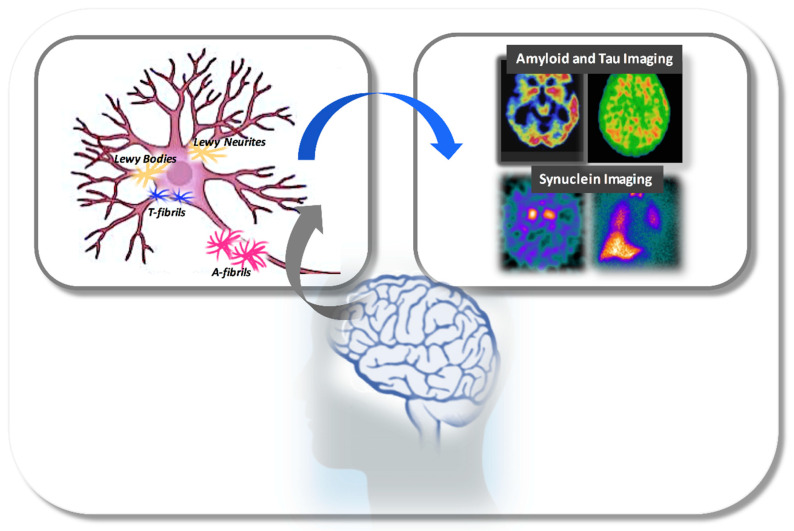
Schematic representation of main neuroaggregates involved in the pathological process of DLB-neurodegeneration and, main imaging tools able to detect. Abbreviations: A-fibrils, Amyloid Fibrils; T-fibrils, Tau-fibrils.

In conclusion, these findings suggest that: (i) when β-amyloid is present, it mainly contributes to a faster temporal lobar atrophy with a pattern of distribution and atrophy resembling that occurring in AD; (ii) when tau is present, cortical aggregates may be associated with cognitive impairment; (iii) when amyloid and tau are present, amyloid-β appears to be associated with cognitive impairment and tau pathology with lower frequency of clinical features of DLB. This may be suggestive of a possible interaction of amyloid, tau and α-synuclein aggregates, which, however, needs further investigation. Imaging approaches mainly used in the early identification of neuroaggregates in DLB neuropathology have been described in Figure 1.

### 7.2. Neuroimaging Biomarkers to Assess Brain Tissue Damage

According to revised DLB consensus criteria, neuroimaging biomarkers to identify structural/functional brain damages have been considered as supportive biomarkers. These are consistent with DLB that help the diagnostic evaluation, but without a clear diagnostic specificity [3].

#### 7.2.1. Structural Damage

The role of structural MRI imaging in the DLB diagnostic criteria is limited. Few cross-sectional and longitudinal MRI studies are present in the existing literature. It is worth pointing out, however, that some authors have recently reported the multivariate studies of structural imaging data in DLB as promising biomarkers [229]. Indeed, the seven studies identified by this systematic review provided preliminary evidence for the capacity of structural MRI biomarkers to differentiate DLB patients from those with AD and healthy controls. The multivariate approaches revealed that in DLB patients, the brain pattern involved the posterior brain areas and subcortical grey matter structures, but mostly sparing the hippocampus [230]. This is in line with previous evidence showing a pattern suggestive of DLB, focused atrophy of the midbrain, hypothalamus, with a relative sparing of the hippocampus and temporoparietal cortex [231]. These findings are also in accordance with the neuropathological ascending model of Lewy Body progression from brainstem to basal areas of the brain [160,231].

It is interesting to note that “pure” DLB is characterized by a relative preservation of the whole brain and medial temporal lobe structures on an MRI scan [3]. However, the coexistence of AD like-neuropathology in DLB resulting in a mixed-neuropathology, can influence this brain pattern of regional atrophy. In this context, some authors have demonstrated that autopsy-confirmed DLB patients without significant AD-type pathology were characterized by lower global and regional rates of atrophy similar to control subjects [205]. In contrast, the mixed DLB/AD patients displayed greater atrophy rates in the whole brain, especially in temporoparietal cortices, hippocampus and amygdala, and ventricle expansion, similar to AD patients [205]. Additionally, they found that in these patients the atrophy rates were correlated with a Braak neurofibrillary tangle stage, cognitive decline, and progression of motor symptoms [205]. The presence of global and regional atrophy rates associated with AD-type pathology in DLB, suggest that these rates can be used as biomarkers of AD progression in patients with Lewy Bodies pathology [205].

#### 7.2.2. Functional Damage

18F-FDG-PET is a very promising neuroimaging tool in the field of the dementias. It reflects the resting state cerebral metabolic rates of glucose, an indicator of regional neuronal activity of specific structures/areas in the brain [232]. Thus, its clinical relevance is related to the possibility to identify the distinct patterns of cerebral glucose metabolism that could help in the differential diagnosis among the dementias [233]. A considerable body of evidence well documents that DLB pattern is characterized by reduced occipital activity and the posterior cingulate island sign on FDG-PET imaging [4]. Indeed, the occipital hypometabolism is currently considered a reliable biomarker for differentiating patients with DLB from those with AD, with a sensitivity and specificity equal to 70% and 74%, respectively [3,234,235].

Notably, the regional metabolic pattern correctly correlates with neuropathology features in DLB-neurodegeneration. Some authors demonstrated that, among widespread cortical regions showing glucose hypometabolism in the DLB group, the metabolic reduction was most pronounced in the visual association cortex compared to that in the AD group [236]. Others have recently determined whether DLB-PET signatures were associated with neuropathology in autopsy-confirmed patients [237]. Their findings demonstrated that among autopsied participants, there was no difference in cingulate island sign or occipital hypometabolism within the Lewy body disease subtypes. By contrast, DLB patients with a lower Braak tangle stage had a higher cingulate island sign involvement compared to those with a higher Braak tangle stage, suggesting a close relationship between neuropathological-clinical features [237].

Considering the clinical relevance of the PET-features, some authors in a multimodal neuroimaging study in vivo focused on the cingulate island sign, investigating associations with medial temporal lobe atrophy. Cingulate island sign refers to sparing of the posterior cingulate relative to the precuneus and cuneus. The rationale of this approach is that the cingulate island sign on PET-imaging is reportedly associated with AD type neurofibrillary tangle NFT pathology in autopsy cases [238]. Indeed, a reduction in cingulate island sign ratio has been reported in association with a high burden of AD-type neurofibrillary tangles [239]. Studies have revealed that the cingulate island sign metabolism is highly specific for detecting DLB, with a specificity of 100% and sensitivity ranging from 62 to 86% [239,240]. In line with this, the cingulate island sign ratio and the degree of atrophy were higher in patients with DLB than in those with AD. These findings suggest that it may be a useful neuroimaging biomarker to evaluate coexisting AD-type NFT pathology [238].

FDG-PET imaging is also highly correlated with the severity and quality of clinical symptoms in neurodegenerative diseases. In particular, it could capture the DLB phenotypic heterogeneity regardless of the “pure” or “mixed” underlying pathology [241]. Some authors recently investigated the brain regions whose metabolic impairment could have specific contributions to the expression of clinical core features in DLB patients. They have evaluated the influence of disease severity on the expression of the general DLB hypometabolic pattern [241]. Results are the following: (i) visual hallucinations and RBD shared a positive covariance with metabolism in the medial temporal lobe, cerebellum, brainstem, basal ganglia, thalami, and orbitofrontal and sensorimotor cortex; (ii) MMSE also positively covaried with metabolism in the left superior frontal gyrus, bilateral–parietal cortex, and left precuneus, and negatively with metabolism in the insula, medial frontal gyrus, hippocampus in the left hemisphere, and right cerebellum. Specific core features were associated with more prominent hypometabolism in specific regions as close clinical–imaging correlation thus reflecting the interplay between topography of neurodegeneration and clinical presentation in DLB patients [241].

### 7.3. Neuroimaging Biomarkers to Assess Neuroinflammation

Neuroinflammation is strongly considered a key contributor to neurodegenerative disorders. In particular, a synergy between microglial activation and tau-pathology has been well-documented in DLB-neurodegeneration. Mounting evidence suggests that tau-pathology may be associated with chronic neuroinflammatory processes including reactive microglia, astrocytes, and increased levels of pro-inflammatory molecules. A possible explanation is that microglia plays an important role in the initiation and progression of tau-pathology and associated neurodegeneration [242].

[11C]-PK11195 positron PET-imaging is a tool able to correctly estimate the microglial activation in the brain. Preliminary data demonstrate the colocalization between [18F]-AV1451 and [11C]-PK11195 tracers, thus suggesting that tau-pathology may have synergistic interactions with neuroinflammation and other processes occurring in DLB [212].

Notably, the involvement of microglia and thus of neuroinflammation appears to be present in early DLB-neurodegeneration. Higher tracer binding, especially in the parietal cortices correlated with widespread lower mean and radial diffusivity, has been detected in DLB patients [243]. Increased microglial activation was associated with a relative preservation of white matter and cognitive functions in DLB, positioning the neuroinflammation as a potential early biomarker of DLB pathogenesis [243]. This is consistent with another study of the same group, in which the authors, by using multimodal imaging and peripheral cytokine analysis, investigated the central and peripheral inflammation in DLB. Neuroinflammation in fronto-temporo-parietal cortices has been reported to be more prominent in DLB subjects with mild cognitive impairment [244]. These findings provide evidence for inflammatory changes in DLB with microglial activation occurring early in the disease, before declining as cognition declines [90].

## 8. Conclusions and Future Perspectives

To the best of our knowledge, this is the first review summarizing and discussing both genetic and neuroimaging biomarkers in the intriguing research field of DLB-neurodegeneration. Our approach might be of great interest, since it allows us to have a broad view on how genetics can influence pathogenetic processes, can determine the accumulation of some neuroaggregates in the brain rather than others and how imaging can detect all these. This is even more important, considering that the same abnormal proteins, different in the burden and pattern of distribution, could result in different proteinopathies and thus in different neurodegenerative diseases. Additionally, in the same disease may coexist “pure” and “mixed” pathologies to which different clinical phenotypes may correspond.

In the last few decades, the identification of DLB as an independent disease together with the development of new omic and imaging technologies has prompted the study of the genetic and epigenetic architecture of the disease as well as the search for biomarkers to be used in the differential diagnosis of the disease with other similar dementias.

Unfortunately, the genetic and epigenetic etiology of the disease is still unknown due to the difficulty in recruiting large cohort of patients with a correct diagnosis of DLB and to the fact that the majority of reported genes/variants linked to the disease have been also associated to different neurodegenerative disorders, or they are often reported in a single study and thus they need to be replicated in future studies.

However, some important data were so far obtained. In particular, it was demonstrated that the potential use of the mutation screening of *GBA* in differential diagnosis of DLB vs. PD and a putative prognostic value of mutations in this gene as demonstrated in Ashkenazi Jewish. Moreover, a possible use of hsa-miR-21-5p and hsa-miR-451 was suggested in the DLB vs. AD differential diagnosis. In the future, we expect an improvement in the knowledge of the genetic and epigenetic role in the pathogenesis of DLB as well as advances in the use of miRNA profiling to perform rapid, standardized and non-invasive differential diagnosis at a molecular level.

The absence of large cohorts to be compared to each other is also due to the reported variability in DLB clinical diagnosis rates between clinicians. A list of criteria has been developed trying to standardize diagnostic processes which also includes the presence of positive biomarkers. Each biomarker and technique has different efficacy in the diagnosis, prognosis and staging of a disease.

Considering the search for biomarkers in biological fluids, unfortunately it was demonstrated that markers specific for DLB are of difficult identification due to its overlap with other diseases. For example, the reduction of GBA, that was reported in DLB independently from the presence of mutations, could be used in DLB diagnosis but it is also a PD feature [245]. However, recently some candidate biomarkers in CSF for differential diagnosis have been proposed, i.e., the GCase activity, VGF, SCG2 and PDYN.

The use of skin biopsies to test pSer129-α-syn deposits is a promising approach in the clinical practice due to its low cost and the unimportance of the biopsy site existing widespread positivity in DLB with autonomic symptoms, but its potential use must be confirmed by additional studies.

Despite all these deficiencies, the existence in DLB of a precious temporal window of several decades in which the characteristic neuropathological changes have already happened but the clinical onset has not, represents a great opportunity for research/clinicians, making them a mine of potential biomarkers. There is convergence that sleep and neurodegeneration are closely associated in a causal rather than casual relationship. Two are possible scenarios to decode this intriguing interaction: (i) sleep disorders cause/accelerate neurodegenerative disorders according to a novel model of a common neurodegeneration; (ii) a common etiology impairs both sleep systems and centres target of neurodegeneration, but in a different time: sleep disorders precede the clinical onset of neurological disorders. This is the case of a REM-parasomnia. Indeed, one of the most important and particular markers of DLB is represented by iRBD that resulted in a valid clinical biomarker of the prodromal DLB stage. Emphasis is placed on the early identification of neuroimaging biomarkers for the opportunity that this creates for neuroprotective treatments and specific disease-modifying therapies.

One of the main strengths of our review is that we propose for the first time a new neuroimaging model to target the pathological neuroaggregates DLB-neurodegenerations. Indeed, we have considered the previous existing evidence on the neuroimaging biomarkers within three sections to target respectively: (i) the neural damage; (ii) the brain tissue damage; (iii) the neuroinflammation. We mainly focused on the molecular imaging able to directly or indirectly bind the neuropathological aggregates in the brain and extra-cranial tissues according to the neuropathological model of the DLB-disease progression.

Several imaging biomarkers with different targets have been tested in DLB. An obvious target in DLB diagnosis is the α-synuclein deposition that can be studied by means of cardiac MIBG scintigraphy or DAT-SPECT. Among these two techniques, the former is a good predictor of clinical conversion of possible to probable DLB, while the latter has the limit to not discriminate DLB form other neurodegenerative disorders. However, a research focus on the development of direct radiotracer of synuclein deposition in the brain and extra-cranial tissue is still mandatory. Additional targets in DLB imaging studies are represented by tau protein and β-amyloid. Amyloid and tau-imaging holds promise as being future biomarkers for differentiating DLB from AD. Multimodal imaging provided a more accurate diagnosis and better treatment of DLB. Multimodal imaging studies that utilize amyloid, tau, and α-synuclein tracers are needed to investigate neurodegenerative changes in DLB.

A great contribution in differential diagnosis could be given by structural/functional imaging that allows not only for differentiating between patients with “pure” DLB from those with AD, but especially to identify AD-like mixed neuropathology. Multivariate data analysis of structural imaging emerges as a promising method for future MRI research in DLB. Atrophy in temporoparietal cortices, hippocampus and amygdala, and the presence of the cingulate island sign on PET imaging were able, by neuroimaging biomarkers, to evaluate the coexisting AD-type NFT pathology in variegate DLB spectrum.

Finally, detecting the microglia activation could represent a target to study the potential initiation and progression of the neurodegenerative process occurring in DLB-neurodegeneration, and thus research focused on this interesting topic should be encouraged. Studies should be still addressed towards the identification of a non-invasive biomarker for predicting DLB before the onset of symptoms. Despite extensive research worldwide, no diagnostic method except for iRBD is currently available for pre-clinical DLB and the existing treatments are only symptomatic.

## Figures and Tables

**Table 1:** Variants and haplotypes linked to DLB *.

	Gene	Detected Variants/ Haplotype	Study/ Inheritance	References
	*APOE*	p.Cys130Arg/ε4/rs429358	GWAS	[14,18,46]
Well-established genes		p.Arg176Cys		
	*APP*	p.Glu599Lys p.Glu674Lys p.Val717Ile Duplication	Rare variants	[20,39,56]
	*GBA*	p.Arg87Gln p.Asp140His p.Arg296Gln p.Glu326Lys p.Glu365 Lys p.Asp448His p.Asn409Ser rs35749011	GWAS	[14,19,41,44]
	*MAPT*	H1G haplotype H2 haplotype p.Gly86Ser p.Ala152Thr p.Arg221Gln	Mendelian	[20,52,53,54,55]
	*PSEN1*	p.Ala79Val p.Gly206Ala p.Glu318Gly	Rare variants	[39,49]
	*PSEN2*	p.Arg71Trp p.Ala85Val p.Val191Glu p.Asp439Ala	Mendelian	[18,49,59]
	*SNCA*	p.Glu46Lys p.Ala53Thr rs7681440 rs356182	GWAS and Mendelian	[14,18,22,60]
Additional genes	*ADGRG7*, *TFG*	chr3:100,357,671–100,439,759	CNV case/controls analysis	[61]
	*ASH1L*	rs12734374	GWAS	[62]
	*BCHE*	K variant	Association study	[63]
	*BCL7C/STX1B*	rs897984	GWAS	[14]
	*CHCHD2*	p.Gly4Arg	Rare variant	[20]
	*CHMP2B*	p.Ile29Val	Mendelian	[46]
	*CHRFAM7A*	2 bp del at 497-498 in exon 6	Association study	[64]
	*CNTN1*	rs7314908	GWAS	[14]
	*CSF1R*	p.Ile794Thr	Mendelian	[65]
	*CSMD1*	chr8:4,033,908–4,126,540	CNV-candidate CNV approach	[61]
	*DCNT1*	p.Ile780Thr	Rare variant	[20]
	*DDX11*, *OVOS2*	chr12:31,249,834–31,407,303	CNV-candidate CNV approach	[61]
	*EIF4G1*	p.Ala502Val p.Gly686Cys p.Met1134Val	Mendelian	[46]
	*GABRB3*	rs1426210	GWAS	[14]
	*GIGYF2*	p.Ser66Thr p.Ser1029Cys	Mendelian	[46]
	*GRN*	p.Cys105Arg p.Ala276Val p.Arg493 *	Rare variants	[20,49,66]
	*LAPTM4B*	chr8:98755,434–98,800,334	CNV case/controls analysis	[61]
	*LPR10*	p.Gly603Arg 1424+5 G→A	Mendelian	[67]
	*LRRK2*	p.Gly2019Ser	Rare variant	[68]
	*MSR1*	chr8:15948,235–16,021,468	CNV case/controls analysis	[61]
	mtDNA	Haplogroup H	Association study	[69]
	*NOS2*	(CCTTT)n	Association study	[70]
	*NME1,NME1-NME2,SPAG9*	chr17:49,177,096–49,231,786	CNV case/controls analysis	[61]
	*NOTCH3*	p.Arg578Cys p.Arg578His p.Arg607His	Rare variants	[20]
	*PARK2*	P.Pro37Leu p.Ala46Ser p.Arg275Trp p.Gly430Asp	Rare variants	[46,49]
	*PDZD2*	chr5:32101,400–32,106,628	CNV case/controls analysis	[61]
	*PINK1*	p.Pro138Leu p.Met318Leu p.Ser499Cys	Rare variants	[49]
	*PRKN*	p.Arg275Trp p.Gly430Asp	Mendelian	[46]
	*PRNP*	p.Met232Arg	Rare variant	[71]
	*SCARB2*	rs6812193	GWAS	[18]
	*SNCB*	p.Val70Met p.Pro123HIs	Mendelian	[29,72]
	*SORL1*	p.Asp140Asn p.Arg1799Gln	Mendelian	[73]
	*SQSTM1*	p.Pro27Leu p.Ala33Val	Mendelian	[46]
	*TBK1*	p.Arg384Trp p.Arg384Gln	Rare variant	[20]
	*TIA1*	p.Pro362Leu	Rare variant	[20]
	*TREM2*	p.Ar62His	Rare variant	[66]
	*ZFPM1*	rs12926163	GWAS	[62]

**Table 2:** Neuroimaging Biomarkers in Lewy Body Dementia (DLB).

Biomarkers Target	Imaging Tool	Assessment	Findings in DLB Patients
**NEURAL DAMAGE**			
**α-synuclein Aggregates**			
**Direct Biomarkers**	[18F]BF227 [18F]WC-58	α-synuclein aggregates load in the brain tissues	High affinity for amyloid low affinity for synuclein Promising affinity for synthetic synuclein fibrils, slow clearance
**Indirect Biomarkers**			
-Noradrenergic System	Cardiac [123I]- MIBG Scintigraphy	Postganglionic presynaptic cardiac sympathetic nerve endings integrity	Cardiac noradrenergic post-ganglionic denervation, expressed as reduced H/R ratio in DLB compared to AD and control subjects
-Dopaminergic System	[123I]-FP-CIT-SPECT	Presynaptic nigrostriatal projection pathway integrity	Dopaminergic neuron degeneration detected as a reduction of radiotracer specific uptake
**-β-Amyloid aggregates**	[11C]PiB PET	Insoluble β-amyloid plaques	Faster temporal lobar atrophy with a pattern of distribution and atrophy resembling that occurring in AD
**-Tau aggregates**	[18F]AV-1451-PET	Intracellular tau in neurofibrillary tangles and neurites	High burden of tau in specific regions including occipital and posterior temporoparietal regions in DLB compared to healthy controls, but less than that observed in AD
**BRAIN TISSUE DAMAGE**			
**-Structural Damage**	MULTIVARIATE DATA ANALYSIS	Atrophy in brain structures/areas	Brain pattern involved the posterior brain areas and subcortical grey matter structures, but mostly sparing the hippocampus
**-Functional Damage**	[18F]-Fludeoxyglucose-PET	Metabolic neuronal activity of specific structures/areas in the brain	Hypometabolism most pronounced in the visual association cortex in DLB compared AD: Cingulate island sign in presence of the coexisting AD-type pathology in DLB
**NEUROINFLAMMATION**			
**-Microglia Activation**	[11C]PK1195-PET	Translocator protein mitochondrial membrane protein in reactive microglia	Higher tracer binding especially in the parietal cortices in early stage of DLB neurodegeneration

Abbreviations: PET, positron emission tomography; SPECT, single-photon emission computed tomography; AD, Alzheimer’s disease; DLB, dementia with Lewy bodies.
